# Priming analogical reasoning with false memories

**DOI:** 10.3758/s13421-015-0513-7

**Published:** 2015-03-18

**Authors:** Mark L. Howe, Sarah R. Garner, Emma Threadgold, Linden J. Ball

**Affiliations:** 1 Department of Psychology, City University London, Northampton Square, London, EC1V 0HB UK; 2 School of Psychology, University of Central Lancashire, Preston, PR1 2HE UK

**Keywords:** Priming, Analogical reasoning, False memory, DRM paradigm, Homonyms, Spreading activation

## Abstract

Like true memories, false memories are capable of priming answers to insight-based problems. Recent research has attempted to extend this paradigm to more advanced problem-solving tasks, including those involving verbal analogical reasoning. However, these experiments are constrained inasmuch as problem solutions could be generated via spreading activation mechanisms (much like false memories themselves) rather than using complex reasoning processes. In three experiments we examined false memory priming of complex analogical reasoning tasks in the absence of simple semantic associations. In Experiment 1, we demonstrated the robustness of false memory priming in analogical reasoning when backward associative strength among the problem terms was eliminated. In Experiments 2a and 2b, we extended these findings by demonstrating priming on newly created *homonym analogies* that can only be solved by inhibiting semantic associations within the analogy. Overall, the findings of the present experiments provide evidence that the efficacy of false memory priming extends to complex analogical reasoning problems.

Memory is highly flexible and reconstructive, designed to retain information about the past, interpret the present, and support simulations of future events (e.g., Howe, 2011; Newman & Lindsay, 2009; Schacter, Guerin, & St. Jacques, 2011). Interestingly, recent research has shown that memory is highly functional, regardless of whether we are talking about memories for events that actually occurred (i.e., *true* memories) or for self-generated memories of events that did not occur (i.e., *false* memories). For example, a significant body of research has demonstrated that true memories are able to prime performance on related memory tasks (e.g., implicit memory; see Gulan & Valerjev, 2010) as well as non-memory tasks such as verbal problem solving (e.g., Mednick, Mednick, & Mednick, 1964).

Priming refers to “a change in the ability to identify, produce, or classify an item as a result of a prior encounter with that item, or a related item” (Schacter, Gallo, & Kensinger, 2007, p. 356). In the case of analogical reasoning, for example, there is a well-established body of evidence demonstrating that people are able to transfer directly their prior memories of problems and their solutions in order to assist them in tackling new, related problems (e.g., Bassok & Holyoak, 1989; Richland, Zur, & Holyoak, 2007; for a recent review, see Holyoak, 2012). Although such analogical reasoning processes appear to rely largely on direct or explicit memory retrieval, there is also evidence that prior memories can influence reasoning and problem solving through intuitive mechanisms that operate indirectly or implicitly. Such intuitive processes appear to have a basis either in tacitly learned memory associations (e.g., Osman & Stavy, 2006; Sloman, 1996) or in rules that were once deliberatively acquired but which have been practiced so extensively that they have reached a state of automaticity in procedural memory (e.g., Kahneman & Klein, 2009).

Kokinov (1990; Kokinov & Petrov, 2001), for example, has shown that implicit memory priming can facilitate performance with complex deductive, inductive, and analogical reasoning problems, benefitting both the strategy taken and the success of the problem-solving process. Schunn and Dunbar (1996) have provided further support for priming effects in analogical problem solving, demonstrating that conceptual knowledge of one knowledge domain (i.e., biochemistry) can spontaneously influence complex reasoning in another, unrelated knowledge domain (i.e., molecular genetics) via implicit priming, leading to facilitated problem solving as measured through both accuracy and the speed of solution generation. Schunn and Dunbar’s sophisticated controls and measures also allowed for any involvement of explicit memory processes to be ruled out as a cause of solution success in the implicit priming conditions.

More recently, it has been discovered that it is not just true memories that can prime performance on cognitive tasks such as problem solving but that false memories can also have key beneficial effects. A common procedure used to generate false memories is the Deese/Roediger-McDermott (DRM) paradigm (Deese, 1959; Roediger & McDermott, 1995). Here, participants are presented with words (e.g., *snooze*, *doze*, *wake*, *rest*) that are all semantic associates of a non-presented word or so-called critical lure (e.g., *sleep*). When asked to remember the words on the list, participants frequently remember the critical lure (a false memory) along with the presented items. Using this paradigm, it has been shown that false memories can prime solutions to problem-solving tasks such as insight-based Compound Remote Associate Tasks (CRATs; see Howe, Garner, Dewhurst, & Ball, 2010) and verbal proportional analogies (Howe, Threadgold, Norbury, Garner, & Ball, 2013).

The latter problems (i.e., verbal proportional analogies) involve the presentation of items that have the form *a* is to *b* as *c* is to *d*, where participants are given the *a*, *b*, and *c* terms and are asked to generate the missing *d* term (e.g., Ball, Hoyle, & Towse, 2010; Goswami, 2001; Goswami & Brown, 1989, 1990). For example, the participant might be given the problem ‘dog is to kennel as bird is to ?’ and asked to generate the solution term. The optimal way to solve such analogies involves identifying the *relation* that exists between the *a* and *b* terms (in this case, ‘inhabits’) and then *mapping* this relation onto the *c* term (‘bird’) in order to generate the answer ‘nest.’ Proportional analogies of this type are non-trivial, especially for children, but even adult performance is rarely error free (e.g., see Green, Fugelsang, & Dunbar, 2006). Such problems therefore frequently feature in intelligence tests (Sternberg, 1977) and in academic examinations such as the statutory assessment test.

Although non-trivial, proportional analogies are typically easier for adults to solve than are many other forms of complex analogy problems that have been studied in the literature, which tend to involve the identification and mapping of multiple, hierarchically embedded ‘systems’ of relations (for pioneering research with such problems, see Gentner & Toupin, 1986; Gick & Holyoak, 1983; Keane, 1987). We acknowledge that proportional analogies do not involve the sophistication of complex analogies of the type that have dominated much of the analogical reasoning literature, and that are typically very challenging for adults to solve in the absence of directive hints to use specific past experiences. Nevertheless, a major advantage of studying false memory priming effects with proportional analogies derives from the way in which such problems afford an opportunity to impose very strict controls on the terms that they are composed of. As will be shown in the experiments that we present below, such controls facilitate the examination of some unique aspects of analogical problem solving that have hitherto remained unexplored.

Although it has been established that both true and false memories can effectively prime solutions to problem-solving tasks (including proportional analogies), an interesting development has been that false memories can actually be more effective primes for problem solving than true memories (Howe et al., 2013; Howe, Wilkinson, & Monaghan, 2012; Wilkinson, 2014). This is consistent with the literature documenting the different strengths of true and false memories where the latter have been shown to be stronger than the former (e.g., Brainerd, Reyna, & Brandse, 1995; Howe, Candel, Otgaar, Malone, & Wimmer, 2010a; McDermott, 1996). For example, whereas true memories decline over time, false memories persist across retention intervals (days, weeks; Brainerd et al., 1995; McDermott, 1996) and negative false memories can actually increase over time (e.g., Howe et al., 2010a).

That false memories can be stronger than true memories has been attributed to the different ways in which they are formed. Specifically, false memories tend to be *self*-*generated* (i.e., occurring spontaneously and automatically as a result of internal semantic activation) whereas true memories are often *other*-*generated* (e.g., presented on a list by the experimenter). This self- versus other-generated difference holds regardless of the nature of the paradigm being used and has been observed using the standard DRM paradigm (e.g., Howe, 2005), when participants are remembering stories, pictures, and videos (e.g., Otgaar, Howe, Peters, Sauerland, & Raymaekers, 2013; Otgaar, Howe, Peters, Smeets, & Moritz, 2014), and when entire memories are being implanted (e.g., Otgaar, Smeets, & Peters, 2012). The efficacy of self-generated information is underpinned by a substantial body of research showing that self-generated information is not only encoded at a deeper level but is also significantly more likely to be remembered than other-generated (i.e., experimenter presented) information (Bjorklund, 2004; Slamecka & Graf, 1978). Thus, if priming effects are monotonically related to memory strength, then false memories should be better primes than true memories, particularly following a delay. The benefit of falsely remembered items in priming solutions to problem-solving tasks (e.g., CRATs) has been established both with adults (Howe et al., 2010b) and children (Howe, Garner, Charlesworth, & Knott, 2011). Howe et al. (2013) attempted to extend this effect to more complex reasoning tasks by using false memories to prime solutions to analogical reasoning problems. Like the research with CRATs, both adults and children were primed on verbal proportional analogies of the form *a* is to *b* as *c* is to *d* and were asked to generate the *d* term. The solution to six of the nine verbal analogies was also the critical lure from previously presented DRM lists (e.g., *desert is to hot as arctic is to cold*, where *cold* was both the solution to the analogy and the critical lure of a DRM list). For the six analogies that were primed (the remaining three were not primed), three were primed by having the critical lure presented as a list item (a ‘true’ or other-generated memory) and the remaining three were primed by not having the critical lure as a list item (a ‘false’ or self-generated memory). The results showed that, unsurprisingly, adults solved the analogies more quickly than children. Importantly, both adults and children solved verbal analogies more quickly when primed with a false memory than when unprimed or when primed by a true memory (there were no differences between the latter two conditions).

Although these effects for false memory priming of analogies are interesting, they are also somewhat limited. This is because Howe et al. (2013) used relatively straightforward analogies that were solved quickly and easily by children and adults alike. Although this allowed for a demonstration of priming effects in both adults and children, a downside is that priming in this context represents activation of simple semantic associates and not the priming of complex reasoning relations themselves.

To explain, the distinction between the priming of simple semantic associations versus the priming of more complex, analytic problem solving is of particular concern in the verbal analogies literature, where a debate exists concerning the mechanisms by which proportional analogies are solved. Some researchers (e.g., Green et al., 2006) suggest that proportional analogies are solved analytically in the optimal manner described above, which involves mapping the relation between the *a* and *b* terms onto the *c* term in order to generate the answer *d*. Others (e.g., Sternberg & Nigro, 1980), however, have proposed that proportional analogies are typically solved using semantic associations (particularly by children; e.g., see Ball et al., 2010; Cheshire, Muldoon, Francis, Lewis, & Ball, 2007; Siegler & Svetina, 2002) in a similar manner to the spreading activation processes thought to underlie the solutions to CRATs. Given the relatively simplistic nature of Howe et al.’s (2013) verbal analogies, solutions could have been generated using associations generated via spreading activation. These analogies could be solved analytically through relational mapping, but given the high semantic association between the *c* and *d* terms, it was equally possible that these analogies provided more of a semantic or word association task than a true test of analogical reasoning. This could mean that what Howe et al. (2013) demonstrated was not the ability of false memories to prime analogical reasoning via a relational-mapping process but simply their ability to prime closely related semantic associations (e.g., where the *b* term ‘hot’ or the *c* term ‘arctic’ simply primed the *d* term ‘cold’). Therefore, a task is needed that can be used to demonstrate the ability of false memories to prime the solutions to complex reasoning problems in the absence of simple semantic associations.

The purpose of the present research was therefore twofold. First, we wanted to develop new analogical reasoning tasks, ones that rely less heavily on simple semantic associations and instead are more dependent on analytic, relational mapping. Second, we wanted to investigate whether false memories are still capable of priming the solutions to these complex analogical reasoning tasks when these solutions rely less heavily on spreading activation among a single set of semantic associations. In order to do this, we have developed two new sets of analogical reasoning tasks.

In Experiment 1, we created a set of verbal proportional analogies that are considerably less semantically related than those used in the previous experiment (Howe et al., 2013). Specifically, by controlling backward associative strength (BAS; a numerical measure of the likelihood that a target word will be produced given a cue word) it was possible to reduce (or in most cases eliminate) the semantic relationship between the *a* to *d*, *b* to *d*, and *c* to *d* terms. We then calculated an overall cumulative BAS score for the target word *d* (solution) being produced as a simple associate of the cue words (*a*, *b*, and *c* terms) provided in the analogy. The lower this cumulative BAS score, the less likely the analogical problem is to be solved by spreading activation of associations in memory from the analogy terms alone, independent of analytic reasoning. When we calculated cumulative BAS for the analogies used in Howe et al. (2013), the value was 3.94. In contrast, the cumulative BAS of the analogies used in our first experiment was 0.23. A one-way analysis of variance (ANOVA) confirmed that there was a significant difference between the cumulative BAS of the analogies in the experiment reported by Howe et al. and those used in our Experiment 1. Thus, Experiment 1 provides a more appropriate demonstration of false memory priming of analogy problems requiring true analogical reasoning rather than problems that merely tap into semantic associations of memory, such as those reported in Howe et al. (2013).

In Experiments 2a and 2b, we extended the priming of analogical reasoning based around an analytic mapping process (as opposed to simple semantic associations) by developing a new type of analogical reasoning task called a *homonym analogy task*. In this task, we used homonyms, which are words that are pronounced the same but have very different contextual meanings (e.g., words such as *score*). In this way we could ensure that analogies were more likely to be solved using analytic mapping of the relational term and not just spreading activation among semantic associations.

## Experiment 1

In Experiment 1, we investigated false memory priming of verbal proportional analogies using a set of normed analogical reasoning problems in which we limited the cumulative BAS of the terms provided in the analogical problem.

### Method

#### Participants

The participants were twenty-five 18-year-old undergraduate students who were fluent in English. Recruitment took place via a participant recruitment system, and each participant received £3.50 for 30 minutes of participation time. Written informed consent was obtained from each participant prior to taking part in the experiment, and participants were debriefed following their participation.

#### Design and materials

A within-participant design was employed consisting of one factor with two levels (Priming: Unprimed or False Memory Priming). The experiment was programmed using Psyscript (an experimental generator) and run by an Apple Macintosh computer. Eight normed proportional analogical reasoning problems (of the format *a* is to *b* as *c* is to *d*) were used in this experiment (see Table 1). These analogies were a subset selected from a previous norming study (Howe, Threadgold, Garner, Bland, & Ball, 2015) in which we asked 50 participants to generate the answers to 50 newly created proportional analogies, with a maximum of 60 seconds being given for the generation of an answer to each problem, after which the correct solution was displayed. Analogies were selected for the present experiment if their normed solution rate fell between 20 % and 80 %, and if the strength of the BAS of the associated DRM list allowed for the attainment of effective experimental controls, as discussed below.

**Table 1:** Mean solution rates and times (with standard deviations in parenthesis) for the normed proportional analogies used in Experiment 1

Analogy with *solution*	Mean Solution rate	Mean solution time (s)
peace : dove :: courage : *lion*	0.76 (.43)	4.56 (3.35)
prevent : restrict :: enable : *allow*	0.74 (.44)	6.02 (3.71)
car : roundabout :: moon : *earth*	0.62 (.49)	5.99 (3.25)
four : cat :: eight : *spider*	0.60 (.49)	5.45 (4.10)
egg : yolk :: plum : *stone*	0.56 (.50)	4.57 (2.32)
wash : clean :: press : *iron*	0.52 (.51)	7.08 (4.62)
leopard : spots :: chest : *hair*	0.46 (.50)	9.31 (6.45)
watch : cog :: compass : *needle*	0.34 .(47)	5.11 (2.43)

The subset of eight analogical reasoning problems that were selected (see Table 1) had normed solution rates in the range of 34 % to 76 %. These eight problems were divided into two groups of four analogies, with the presentation of these groups being counterbalanced across participants in the experiment in terms of whether they were unprimed or primed by the prior presentation of a DRM list. The four analogies in each group were equated on the BAS of the DRM list items and on their normed solution rates (Group 1 analogies—*earth*, *lion*, *stone*, *iron*: Mean DRM BAS = .20, Mean Solution Rate = 61.5 %; Group 2 analogies - *allow*, *spider*, *needle*, *hair*: Mean DRM BAS = .14, Mean Solution Rate = 53.5 %). Because BAS is a widely used measure of the strength of a DRM list in producing false memories, it is important to control for Mean DRM BAS level across conditions (e.g. Roediger, Watson, McDermott, & Gallo, 2001).

Furthermore, to provide an indication of the semantic strength of the analogy the BAS of the *a* to *d*, *b* to *d*, and *c* to *d* relationships (e.g., the likelihood of producing the solution *d* when asked to provide a semantic associate of *a*, *b*, and *c*) were totaled, providing a cumulative BAS score for each analogical problem. All BAS values were selected from the normed associates presented by Nelson, McEvoy, and Schreiber (1998). There was no significant difference in the cumulative BAS for the *a* to *d*, *b* to *d*, and *c* to *d* relationships between each group of four analogies (Cumulative BAS: Group 1 = .06, Group 2 = .17, *p* > .05). All eight analogies had a zero BAS score for the *a* to *d* and *b* to *d* relationships. Three analogies (those with the solutions *iron*, *hair*, and *needle*) had an above zero, but still very low, *c* to *d* BAS score (.06, .14, and .03, respectively). Overall, then, there was a very low likelihood of the *a* and *b* cue words in any of the analogies producing solution words by spreading activation alone.

For each analogical reasoning problem there was a linked DRM list consisting of 12 associated words where the critical lure was also the solution to the problem (see Appendix A for DRM lists and BAS scores for each list). DRM lists containing 12 associate terms were used. The use of 12 associates is consistent with early applications of the DRM paradigm (Deese, 1959) and has been frequently shown to induce false recall of the critical lure (Roediger & McDermott, 1995). The DRM lists were either selected from standard sources (e.g., Roediger et al., 2001) or were constructed based on the normed associates presented by Nelson et al. (1998). Words on the DRM lists did not appear as part of the analogical reasoning problems.

#### Procedure

Participants were informed that they would be completing two distinct tasks: a memory task and problem-solving task. Therefore, it was never explicitly stated to participants that the word lists were linked to the analogical reasoning problems in any way, or that the memory task could be used to help solve the analogical reasoning problems.^1^ Participants initially listened to four DRM lists played to them through headphones via a computer. DRM items were presented at the rate of one word every two seconds. Lists were played individually and in a random order for each participant, but the order of the items in the list remained constant for each participant. Following presentation of a list there was a brief filler task (consisting of two simple arithmetic calculations on screen) before participants were asked to write down as many words as they could remember on a piece of paper, provided. Participants were not given a time limit to recall these words and were merely instructed to proceed when they had recalled as many words as they could from the given list. Following the completion of all four DRM lists, participants were asked to turn their paper over so they could not see their recall answers before attempting to solve eight analogical problems, presented one at a time to them on the computer screen.

Presentation of the eight analogical problems was randomized for each participant. Analogical problems were presented in the format ‘*a* is to *b* as *c* is to _____’ in the center of the computer screen. Participants were required to click a button as soon as they had their final answer to an analogy, and they then needed to type their answer into the space provided on screen. The timer began as soon as participants viewed the analogy on screen and ended once participants had clicked the button signaling that they had their final answer. Participants received a maximum of 60 seconds to generate each answer, after which the correct answer was displayed. On providing their answer to each analogy, participants viewed the complete analogy with the correct answer on screen. Participants completed eight analogies in total, four of which had been primed by the associated DRM list and four of which were unprimed.

### Results

The mean analogy solution rate (proportion) and the mean analogy solution time (seconds) were calculated for each participant and analyzed in separate ANOVAs. For the primed analogical reasoning problems, solution rates and times were further conditionalized according to whether a false memory had been produced during recall of the DRM list relevant to that analogy. Conditionalizing primed performance in this manner has been widely used in previous research investigating the priming capacity of false memories (e.g., Howe et al., 2010b, 2013). Despite the reduction in items per cell when responses are conditionalized (although there was still sufficient power to detect differences should they exist as the majority of participants, over 65 %, contributed data to all three cells), it is imperative that this distinction is made because previous research has consistently shown that priming is only effective when the false memory is actually produced at on a memory test. Therefore, there were three levels of priming for the analyses that we report below: unprimed vs. false memory primed with no false recall vs. false memory primed with false recall. The mean false memory proportion was .26 (*SD* = .13) with the majority of participants (84 %) having one or more false memories.

#### Solution rates

**Fig. 1:**
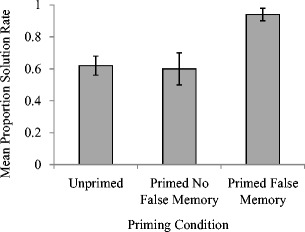
Mean proportion of solution rates (with standard errors) as a function of priming condition

There was a significant main effect of priming for solution rates, *F*(2, 24) = 6.17, *p* < .05, *η* ^2^_p_ = .34. As can be seen in Fig. 1, and which was confirmed using post hoc pairwise comparisons, solution rates were significantly higher in the false memory priming condition when a false memory had been produced at recall (*M* = .94, *SE* = .04) compared to either false memory priming where no false memory was produced (*M* = .60, *SE* = .02, *p* < .05) or the unprimed condition (*M* = .62, *SE* = .06, *p* < .05). There was no significant difference in solution rates between the latter two conditions (*p* > .05).

#### Solution times

**Fig. 2:**
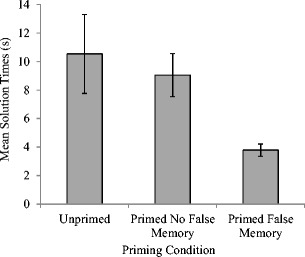
Mean solution times (seconds) with standard errors as a function of priming condition

Like the solution rate data, there was a significant main effect of priming for solution times, *F*(2, 20) = 4.72, *p* < .05, *η* ^2^_p_ = .32. As can be seen in Fig. 2, and which was confirmed using post hoc pairwise comparisons, solution times were significantly faster in the false memory priming condition when a false memory had been produced (*M* = 3.79 s, *SE* = .43) compared to either the false memory priming with no false memory (*M* = 9.05 s, *SE* = 1.51, *p* < .05) or the unprimed conditions (*M* = 10.54 s, *SE* = 2.77, *p* < .05). There was no significant difference between solution times in these latter two conditions (*p* > .05).

### Discussion

The results of Experiment 1 are unique inasmuch as they show that having a false memory is critical for the priming of analogical reasoning that requires a relational mapping process to arrive at a solution. That is, only when participants recalled the critical lure did false memories prime the solutions to verbal proportional analogies. When participants failed to recall the critical lure, performance (solution rates and solution times) on analogical reasoning problems was no better than when solutions had not been primed. Thus, we can conclude that the production of the critical lure is imperative for the success of a false memory priming effect in analogical reasoning.

These findings also provide an important and unique demonstration of the benefit of false memories, one that extends our knowledge of their ability to prime performance not just on related memory tasks but on more complex problem-solving tasks as well. Although previous research has been influential in establishing evidence for the ability of false memories to prime the solutions to insight problems (e.g., Howe et al., 2010b, 2011), this is the first experiment to extend these findings to more complex analogical reasoning tasks, ones that require a process of analogical mapping, and that cannot be solved solely by activating simple spreading activation among semantic associations (as was the case in Howe et al., 2013).

Making this distinction is particularly important for theories of analogical reasoning, where a debate exists concerning the mechanisms by which proportional analogies are solved. Here, some researchers argue that analogies are solved by a process of semantic association and not by using analogical mapping. The results of the present experiment suggest that when one limits the availability of semantic associations between the analogy terms and the solution, it is still possible both to solve these analogies (60 % of the time) and, importantly, to prime these solutions using false memories.

## Experiment 2a

Experiment 1 utilized a set of normed verbal proportional analogies in which we limited the likelihood of the target solution being arrived at using spreading activation through semantic associates. However, despite this control, one could argue that this association, rather than being removed, was simply more remote than in the analogies used previously by Howe at al. (2013). That is, the analogies presented in Experiment 1 might still have been solved via spreading activation, albeit requiring the activation of more distant or weaker associations (for how such a process might work, see Nelson, Kitto, Galea, McEvoy, & Bruza, 2013; Nelson, McEvoy, & Pointer, 2003).

If semantic association did still play a role in solving analogical reasoning problems in Experiment 1, what would happen if we were to remove the influence of associations by ensuring that the proportional analogy could only be solved via an analytic mapping process? Moreover, what would happen if analogical problem solving purposefully required participants to inhibit any semantic associations that may be apparent within the proportional analogy?

In order to examine these questions, we developed a task that not only removed the use of semantic association as a solution strategy but also required the inhibition of dominant semantic associations within the analogy problem in order obtain the solution using an analytic mapping process. We did this by creating a new type of verbal proportional analogy termed a *homonym analogy*. Homonym analogies take the standard form of a verbal proportional analogy, that is, *a* is to *b* as *c* is to *d*. For example, ‘*fur* is to *bear* as *bark* is to *tree*.’ However, these analogies differ in a number of important ways to the standard verbal proportional analogies used in Experiment 1.

To see how these homonym analogies work, consider the following example: *fur* is to *bear*, as *bark* is to: __? Rather than have participants solve these analogies in the usual manner, we gave participants four multiple-choice options, in this case, *branch*, *dog*, *meow*, and *tree* (see Table 2). In this example, the *a* and *b* terms, *fur* and *bear*, create a context related to the category of animals, making one likely to use this context when interpreting the ambiguous homonym *c* term, *bark*. If participants are biased by this context, they will interpret the *c*-term *bark* in terms of an animal (the noise a dog makes) and select the answer *dog*. In the multiple-choice options, we included two incorrect but contextually relevant associates (one high associate and one low associate: *dog* and *meow*, respectively) to determine whether participants are biased towards using semantic associations rather than relational mapping to solve these problems. Alternatively, when given the multiple-choice options, if one reasons correctly that *fur* is the outside of a *bear*, one will apply this relation analytically to the *c* term, the homonym *bark*, and correctly reason the solution that *bark* is the outside of a *tree*. A further associate of this correct context is also provided, other than the correct answer, which in this example is the word *branch*. If participants incorrectly select this solution word during the task it would suggest that although they are able to inhibit the incorrect meaning of the homonym (or even interpret the homonym with the required meaning to solve the problem, without any consideration of the *a* and *b* terms) they might still reason incorrectly.

**Table 2:** Multiple choice answers for the homonym analogy ‘*fur* is to *bear*, as *bark* is to *tree*.’

Multiple choice answers	Example item
Correct answer	Tree
Correct context associate	Branch
Incorrect context high associate	Dog
Incorrect context low associate	Meow

In summary, there are a number of critical differences between standard proportional analogies and our newly created homonym analogies. The first important difference is that the *c* term used in these new analogies is a homonym, that is, a term that can have multiple meanings in different contexts and which is therefore ambiguous in nature. Second, the *a* and *b* terms in a given homonym analogy set a context related to one of the meanings of the *c* term, specifically, a context that is *not* related to the solution to the analogy. Third, the *d* term used to solve the analogy requires participants to inhibit the context created by the *a* and *b* terms and to access the alternative meaning of the *c* term in order to achieve the solution.

A final difference is that participants are asked to select the analogy solution from among four multiple-choice options. This is a deviation from the methodology employed in Experiment 1, in which participants were asked to generate the *d* term response to standard verbal proportional analogies. A multiple-choice response paradigm was adopted with the homonym analogies so that it was possible to analyze specific types of errors provided to these problems by participants. The options consisted of the correct solution and three incorrect choices that were carefully selected to fall into one of three categories: (1) a *correct context associate* – a term that is semantically related to the correct solution, which also requires participants to access the correct meaning of the homonym; (2) an *incorrect context high associate* – a term that is highly semantically associated to the homonym when taken in the context of the *a* and *b* terms of the analogy, but which is incorrect when one achieves an effective relational mapping from the *a* and *b* terms to the *c* term; and (3) an *incorrect context low associate* – a term that is a low semantic associate of the homonym when taken in the context of the *a* and *b* terms of the analogy, but which is again incorrect when one achieves an effective relational mapping from the *a* and *b* terms to the *c* term.

When constructing the multiple choice items from which participants selected their final answer, written word frequency was controlled using the Kucera-Francis written word frequency scores obtained from the MRC Psycholinguistic Database (Coltheart, 1981). A highest word frequency item from the four that were presented occurred as a critical item and the incorrect context high associate on three instances, and as a correct context associate and incorrect context low associate on two instances each. Therefore, any multiple choice answer type (see Table 2 for examples) was not likely to be consistently selected based on dominant written-word frequency alone.

Given the multiple contextual interpretations of homonym terms, it is important to consider the dominance of any single homonym context in comparison to its counterpart meanings. To do this, we consulted Twilley, Dixon, Taylor, and Clark’s (1994) frequency norms for the different meanings of our critical homonym *c* terms. Drawing on the previously utilized analogy example with the homonym *bark*, Twilley et al. (1994) noted the primary context is in terms of *dog* and secondarily in terms of *tree*. Therefore, the overall analogy is consistent with the dominant homonym context (animals or dog), while the correct answer context (bark in terms of the outer lining of a tree) is consistent with the second dominant meaning of the homonym. We consider the effects of homonym dominance in terms of responses to homonym analogies with Experiment 2a. In Experiment 2a, we collected norms for these new types of problems by having participants solve a set of homonym analogies.

### Method

#### Participants

Fifty-six participants (9 males; 47 females) aged 16 to 18 years old took part in this experiment. All participants aged 18 and over provided written informed consent prior to the experiment, and for those aged 16 or 17 years, parental consent was sought prior to participation. At the end of the experiment, all participants were fully debriefed about the purpose of the experiment.

#### Design

A within-participant design was used, with each participant completing a selection of three out of nine homonym analogies. The order of analogies was randomized. Because the answer to the homonym analogy is the opposite context to that presented in the analogy, this may have become apparent to participants after solving a number of them. Participants were therefore asked to complete three out of nine analogies in order to prevent practice effects at solving these problems. This issue was addressed in Experiment 2b by the introduction of non-homonym analogies similar to those used in Experiment 1, which served as distractor problems.

#### Materials and procedure

The analogical reasoning problems utilized in this experiment were the newly formed homonym analogies. Nine such analogies were created (see Appendix B). For each analogical reasoning problem participants were provided with four possible answers to choose from. One of these was the correct solution, and then there were also three possible foils: a correct context associate, an incorrect context high associate, and an incorrect context low associate.

The procedure was identical to the problem-solving component in Experiment 1, with the exception that participants completed three of nine homonym analogies. Furthermore, participants were asked to choose an answer from one of four provided. These options were displayed directly underneath the analogy and labeled *a* to *d*. The position of the correct answer in terms of *a* to *d* was randomized.

### Results

The percentage of participants solving each homonym analogy within the time limit was calculated along with mean solution times. Overall, solution rates for homonym analogies were at .68 (*SD* = .26) and solution times averaged 10.54 s (*SD* = 6.07). Table 3 shows the proportion correct per analogy. In addition to solution times and rates, we were interested in the types of errors participants made on these new homonym analogies. In particular, we were interested in which of three incorrect choices participants made when they were unable to solve the analogy correctly and how quickly they made these errors. Errors were categorized into one of three possible types for each analogy based on their selection on the multiple-choice portion of the task. Errors were defined as correct context associate errors, incorrect context high associate errors, or incorrect context low associate errors (see Table 4).

**Table 3:** Proportion correct (standard deviations in parenthesis) for each homonym analogy

Analogy (*solution*)	Proportion correct
run : legs :: stitch : *needle*	.79 (.42)
table : surgery :: organ : *music*	.36 (.49)
roar : lion :: horn : *car*	.79 (.42)
weapon : gun :: bug : *spider*	.88 (.32)
vowel : letter :: capital : *city*	.83 (.39)
engrave : wood :: log : *book*	.41 (.51)
alive : dead :: wake : *sleep*	.95 (.22)
February : month :: date : *fruit*	.50 (.51)
starve : eat :: fast : *slow*	.60 (.50)

**Table 4:** Proportion of errors (standard deviations in parenthesis) for each homonym analogy by error type

Analogy (*solution*)	Correct context associate	Incorrect context high associate	Incorrect context low associate
run : legs :: stitch : *needle*	.36 (.49)	.41 (.50)	.24 (.43)
table : surgery :: organ : *music*	.13 (.34)	.70 (.46)	.17 (.37)
roar : lion :: horn : *car*	0	1 (0)	0
weapon : gun :: bug : *spider*	.41 (.50)	.53 (.51)	.06 (.24)
vowel : letter :: capital : *city*	.09 (.30)	.81 (.40)	.09 (.30)
engrave : wood :: log : *book*	.15 (.39)	.73 (.45)	.12 (36)
alive : dead :: wake : *sleep*	0	.1 (.57)	0
February : month :: date : *fruit*	0	.87 (.33)	.12 (.33)
starve : eat :: fast : *slow*	.52 (.51)	.24 (.44)	.24 (.44)

A one-way ANOVA for error type (correct context associate vs. incorrect context high associate vs. incorrect context low associate) was conducted on the proportion of each error selected for the analogies. A significant main effect was found, with post hoc pairwise comparisons revealing that significantly more incorrect context high associate errors were made (*M* = .5, *SD* = .48) than correct context associate errors (*M* = .14, *SD* = .33) or incorrect context low associate errors (*M* = .11, *SD* = .29), with the latter two not differing significantly from one another, *F*(2,110) = 14.62, *p* < .01, *η* ^2^_p_ = .28.

For solution times, we examined whether participants differed between correctly and incorrectly solved analogies. A paired *t* test revealed that solution times for correct answers (*M* = 10.30s, *SD* = 5.02) were significantly faster than solution times for errors (*M* = 15.00s, *SD* = 10.27), *t*(41) = -3.25, *p* < .01.

Given that the homonyms used in the *c* position of the analogy all have at least two (and sometimes three or four) contextual meanings, it is important to consider whether the ordinarily dominant context (regardless of the contextual interpretation of the *a* and *b* terms in the analogy) influences the types of answers selected, whether correct or incorrect. Using the Twilley et al. (1994) norms, we obtained dominance ratings for the context of each homonym (*c* term) in terms of whether the incorrect analogy context afforded by the *a* to *b* terms was consistent with the normally dominant interpretation of the homonym, or whether the analogy answer (i.e., the correct context) was consistent with the dominant interpretation. Taking the analogies presented in Table 3, for six of the nine analogies the context of the correct answer was also the dominant context of the homonym (analogy solutions—*spider*, *slow*, *needle*, *city*, *car*, *sleep*), whereas for the remaining three analogies (analogy solutions—*fruit*, *music*, *book*) the incorrect analogy context established by the *a* and *b* terms was the dominant context (or a more dominant context) than the answer context of the homonym. It was not possible to have an even division of homonym analogies in which the correct or incorrect context was the dominant context of the homonym due to the constraints of material design to obtain accurate homonym analogies with appropriate DRM lists. As such, it was decided that the majority of analogies should be within the category where the dominant context was the answer context.

Given this, the question arises as to whether it is easier to access the analogy solution when the required context for the answer is also known to be the most dominant context of the homonym (as determined by norms established by Twilley et al., 1994). Although we cannot directly compare across responses because participants completed three of the nine analogies, it is possible to provide mean solution rates and times for the analogies. The mean solution rates and times indicated that when the answer to the analogy was consistent with the dominant context of the homonym, participants seemed to access the answer more readily (*M* = 10.52 s), and with greater accuracy (*M* = .81) in comparison to when the context established by the *a* and *b* analogy terms was more dominant (*M* = 12.43 s, *M* = .43, respectively). This suggests that homonym dominance might make the answer easier to access, and the *a*, *b*, and *c* terms of the analogy somewhat easier to inhibit, if the answer is consistent with the dominant homonym interpretation.

If we take the correct context errors and incorrect context high associate errors and look at these in terms of homonym dominance, it would seem as if participants made, on average, more incorrect context high associate errors when this incorrect context was the dominant context of the homonym (*M* = .71) in comparison to when the answer or correct context was dominant (*M* = .63). If we look at the correct context associate errors, participants make more of these particular errors when the answer context (correct context) was dominant (*M* = .16) than when the context established by the *a* and *b* analogy terms (incorrect context) was dominant (*M* = .08). Of course, these effects have to be interpreted with caution, given the overall low rate of correct context response errors overall compared to incorrect context high associate errors, regardless of homonym dominance. However, the fact remains that homonym dominance can have an influence on response errors inasmuch as errors tend to be consistent with the dominant interpretation of the homonym.

### Discussion

The results of Experiment 2a provide evidence that errors made while solving homonym analogies are through a bias towards selecting the highest semantic associate to the *c* term in the analogy, even when this item is incorrect. When a homonym analogy was solved incorrectly, participants were significantly more likely to have selected the highest semantic associate of the incorrect context (i.e., the semantic associate of *c* interpreting the homonym in the context set by the *a* and *b* terms of the analogy), rather than a lower associate of the incorrect context, or a semantic associate of the correct context.

The tendency toward selecting the highest semantic associate of the *c* term (in the context established by the *a* and *b* items) during an error response, suggests a bias towards selecting a high semantic associate of *c*, even when this item is not the correct one when solved by an analytic process of relational mapping. The propensity to be drawn toward solving verbal proportional analogies by semantic association is well established, particularly in terms of how children solve these analogies (Sternberg & Nigro, 1980; see also Ball et al., 2010; Cheshire et al., 2007; Siegler & Svetina, 2002). However, it is widely believed that adults utilize a more sophisticated process of relational mapping to arrive at the correct answer (Green et al., 2006). In contrast, what the current analysis of the errors made during the solving of homonym analogies suggests is that adults are also drawn to a high semantic associate of the *c* term when solving verbal proportional analogies. One possibility is that those making errors use semantic association as a heuristic to aid in selecting the solution, rather than identifying the relationship between the initial two analogy terms, and applying this to the latter part of the analogy. In other words, people may be defaulting to the use of semantic association rather than reasoning by means of an analytic mapping process based on the relation that exists between the *a* and *b* terms within the analogy. If this is the case, we would expect that participants who are drawn towards making an incorrect context high or low associate error would also solve analogies faster than those who solve the analogies correctly.

Previous standard forms of analogical reasoning problems have rendered it difficult, if not impossible, to distinguish between the correct solution strategy of relational mapping (Green et al., 2006) versus the potentially incorrect method of solving by semantic association. This is because typical verbal analogical problems are often confounded by the fact that the *c* and *d* terms not only have a relational link but are also often highly semantically associated (Howe et al., 2013). For example, in the problem ‘*pyramid* is to *cube* as *triangle* is to *square*,’ triangle and square are highly semantically associated, and participants might be likely to generate square in the absence of analogical reasoning. The use of our newly designed homonym analogies demonstrates that the application of this heuristic by adults can lead participants to arrive at an incorrect solution. Indeed, the current findings suggest that the context of the analogy is important in participants’ overall decision when selecting a solution, such that participants are often drawn towards an incorrect answer that fits with the context of the homonym established by the *a* and *b* terms, rather than the alternate context established by the *c* term. Thus, the overall context and the relation to the semantic association of *c* might be important in solving typical analogies.

Furthermore, responses to homonym analogies can be influenced by the dominant context of the homonym term. However, even when the dominant context is the correct answer, it can be difficult to overcome the context set by the analogy terms. Homonym dominance can lead to errors consistent with the dominant interpretation of the homonym. For the majority (six out of the nine) of the homonym analogies presented in Experiment 2a, the dominant context was the answer context (e.g., the ‘correct context’), yet participants were still drawn to making incorrect context associate errors when solving homonym analogies (and were biased by the context provided in the analogy terms), even when this was *not* the dominant context of that homonym. Despite the fact that these analogy problems seem to be solved more accurately when the analogy solution context was the dominant interpretation, it still did not prevent participants from making incorrect context response errors. That is, participants’ responses may be biased by the incorrect context established by the *a* and *b* terms of the analogy even when this context is a less dominant than the correct context. Given that accuracy rates for the analogies in which the correct context is dominant were far from ceiling, and that there were significantly more incorrect context errors in the set of analogies where this incorrect context was not a dominant interpretation, homonym dominance does not entirely determine response selection. Indeed, participants were mainly influenced by the context of the *a* and *b* analogy terms when interpreting the *c* term and not simply relying on their existing knowledge base of homonym context interpretations.

The use of homonym analogies demonstrates that errors are made when participants have a bias to generate the solution term in the context of the *a* and *b* components of the analogy such that they then search for a similar semantic associate of *c*. The correct solution to a homonym analogy—one that involves the *a*–*b* relation—necessitates inhibition of not only the context provided by the *a* and *b* terms, but also of the highest semantic associate of *c* to this context. Research has demonstrated that young children often struggle with inhibitory control in analogy problems (e.g., Richland, Morrison, & Holyoak, 2006). Experiment 2a provided evidence that errors made by adult participants in analogical reasoning with homonym problems can also arise from difficulty in inhibiting the context of the analogy and the automatic spreading activation to semantic associates of *c* to this context.

## Experiment 2b

The aim of Experiment 2b was to ascertain if false memory priming can help adults overcome the bias observed in Experiment 2a, whereby they tend to generate the solution to a proportional analogy by searching for a semantic associate of *c* in the context established by the *a* and *b* terms of the analogy. Generating a false memory at recall would be expected to make this item more salient in memory, thereby priming the availability of this item as a solution term during subsequent analogical reasoning. We therefore expected that false memory priming would benefit participants in that they would be able to inhibit the tendency to use the heuristic in the analogy that leads to the incorrect answer, that is, simply generating a high semantic associate of *c* in terms of the (incorrect) context that is established by the *a* and *b* terms of the analogy.

### Method

#### Participants

A total of 46 females aged 18 years participated in the experiment. Each participant provided written informed consent prior to taking part in the experiment, and participants were fully debriefed at the end. All participants were fluent in English.

#### Design and materials

We employed a within-participant design similar to Experiment 1. This consisted of one factor with two levels (Priming: Unprimed or False Memory Primed). The experiment was programmed using Psyscript and played by an Apple Macintosh computer. Thirteen analogies were used in this experiment (see Appendix C). Of the 13 problems, 10 critical analogies were designed such that the *c* term within the analogy was a homonym term (e.g., *bark* as ‘the noise a dog makes’ or, consistent with the *a* is to *b* relation in the analogy, *bark* as ‘the outer lining of a tree’). The other three analogical problems were included to form distractor items. Three distractor items were included in the set of solved analogical problems to ensure that participants did not identify a consistent pattern within the homonym analogies such that the answer was always the opposite context to the terms presented within the analogy. One of these three was also a homonym analogy, while the remaining analogies were non-homonym analogies similar to those used in Experiment 1 (see Appendix A). The 10 critical analogical problems (see Appendix C) were divided into two groups equated on the BAS of the DRM list items associated with each analogy problem (Group 1: Mean BAS = .33, Group 2: Mean BAS = .24). In Group 1, four out of five analogies had the dominant interpretation of the homonym as the correct context; in Group 2, this was three out of five analogies.

For each analogical problem, participants were presented with a choice of four items from which to select their answer. Only one of these answers was the correct answer. For the 10 critical analogies, the three alternative responses were composed of an associate of the correct answer and two associates of an incorrect context answer.

For each analogical problem there was a linked DRM list consisting of 12 associated words where the critical lure was the problem solution (refer to Appendix C for the DRM lists and the associated BAS scores). DRM-list words that overlapped with the items presented in the analogical problems were removed so that DRM items were not presented as part of any subsequent analogy items. The single exception to this was the word *fast*, which was integral to the DRM list *slow* and was therefore left in the list. DRM lists were selected such that they only primed one context (refer to Appendix C for DRM lists). Presentation of the materials was counterbalanced such that each analogy group served in the unprimed and primed conditions.

#### Procedure

The procedure was identical to that of Experiment 1 except that participants completed 13 analogies rather than eight. Furthermore, participants were asked to choose an answer from one of four provided. These options were displayed directly beneath the analogy, and were labeled *a* to *d*. The position of the correct answer was randomized across participants.

### Results

The mean analogy solution rate (proportion) and the mean analogy solution time (seconds) were calculated for each participant. Solution rates and times were analyzed separately in two analyses of variance (ANOVAs). For the primed analogy problems, solution rates and times were further conditionalized according to whether the participant produced the critical lure item (i.e., primed and produced a false memory) or did not (i.e., primed but did not produce a false memory). Therefore, like Experiment 1, for the purposes of the analyses there were three priming conditions (i.e. unprimed vs. primed with no false memory vs. primed with false memory recalled). Like Experiment 1, the majority of participants (over 75 %) contributed data to all three cells. The mean false memory proportion was .34 (*SD* = .21), with the majority of participants (78 %) having one or more false memories.

#### Solution rates

**Fig. 3:**
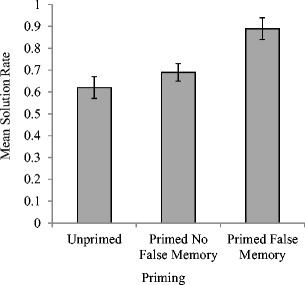
Mean proportion of solution rates (with standard errors) as a function of priming condition

There was a significant main effect of priming on solution rates, *F*(2, 58) = 10.3, *p* < .001, *η* ^2^_p_ = .26. As can be seen in Fig. 3, and which was confirmed using post hoc pairwise comparisons, solution rates were significantly higher in the false memory condition when a critical lure had been produced (*M* = .89, *SE* = .05) in comparison to either false memory priming where no critical lure was produced during recall (*M* = .69, *SE* = .04, *p* < .05) or the unprimed condition (*M* = .62, *SE* = .05, *p* < .05). No significant difference was found between the latter two conditions (*p* > .05). Figure 3 displays solution rates for each condition.^2^

#### Solution times

There was no significant main effect of priming on solution times, *F*(2, 56) = .659, *p* > .05. Analogical problem solutions were solved equally fast when unprimed (*M* = 11.31 s, *SE* = 1.03), primed with no false memory (*M* = 11.87 s, *SE* = .94), or primed with false recall of the critical lure (*M* = 10.56 s, *SE* = 10.57). However, Mauchly’s test revealed that the assumption of sphericity had been violated for the solution time data (*p* = .02), indicating that there was considerable variability across participants in solution times. Furthermore, examination of a histogram suggested that the solution time data were bimodally distributed, in that there were two groups of solution times, reflecting participants who were *fast solvers* and participants who were *slow solvers*.

Because of the bimodal distribution, we decided to examine solution times separately for fast solvers and slow solvers by splitting participants on the basis of their mean solution times for unprimed analogies. This method of splitting solution time data into two groups of fast and slow solvers is consistent with that described by Garner and Howe (2014) when analyzing solution times for CRAT problems. In what follows, we describe analyses that included the addition of *group* (fast vs. slow solvers) as a post hoc between-participants factor.

##### Comparing fast and slow problem solvers

**Fig. 4:**
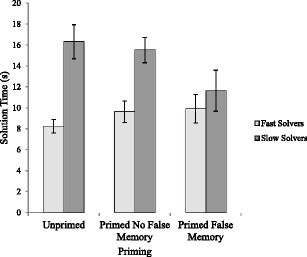
Mean solution times (s) with standard errors for fast and slow solvers as a function of priming condition

It should be noted that a one-way ANOVA on solution rates revealed a marginally significant difference between the fast and slow solvers, *F*(1, 45) = 4.189, *p* =.047, where fast solvers were slightly less accurate (*M* = .62) than slow solvers (*M* = .71) in their responses. This suggests that fast responding might generate a speed accuracy trade-off. For the solution times, a 2 × 3 mixed ANOVA (Group x Priming) was conducted with group as the between-participants factors with two levels (fast vs. slow solvers) and the within-participant factor of priming with three levels (unprimed vs. primed no false memory vs. primed with false memory). Not unexpectedly, the results showed that there was a significant main effect of group, *F*(1, 27) = 323.13, *p* <.001, *η* ^2^_p_ = .93, with the fast solvers group solving the analogical problems significantly more quickly (*M* = 9.27 s, *SE* = .81) than the slow solvers group (*M* = 14.49 s, *SE* = 1.04). As before, there was no main effect of priming, *F*(2, 54) = 1.51, *p* >.05, with problems being solved equally quickly in the unprimed (*M* = 11.49 s, *SE* = .81), primed with no false memory (*M* = 12.06 s, *SE* = .78), and primed with false memory (*M* = 10.25 s, *SE* = 1.00). However, consistent with our intuition (and Garner & Howe’s, 2014, previous CRAT findings), there was a Group x Priming interaction, *F*(2, 54) = 4.19, *p* <.05, *η* ^2^_p_ = .13 (see Fig. 4).^3^

To ascertain the source of the significant interaction we employed simple main effects analyses using a Bonferroni correction for multiple comparisons. There was a significant difference between fast and slow solvers in the unprimed condition (*p* < .001) such that fast solvers completed the unprimed analogical problems significantly faster (*M* = 8.24 s, *SE* = .64) than the slow solvers (*M* = 16.32 s, *SE* = 1.18). Fast solvers (*M* = 9.64 s, *SE* = 1.01) also solved the problems significantly faster than slow solvers (*M* = 15.52 s, *SE* = 1.28) when primed with no false memory (*p* < .001). However, there was no significant difference between fast solvers (*M* = 9.91 s, *SE* = 1.42) and slow solvers (*M* = 11.63 s, *SE* = 1.82) in the primed with a false memory condition (*p* > .05). This indicates that for participants who were primed and who produced a false memory at recall, the priming effect benefits the slow solvers (i.e., they were as fast as the fast solvers) perhaps because the fast solvers are already at ceiling for solution times.

### Discussion

The findings from Experiment 2b demonstrate that false memory priming of homonym analogy problems leads to significantly higher solution rates than when those same problems are unprimed or are primed but no false memories are generated at recall. Moreover, the results of Experiments 2a and 2b show that when participants make errors in solving homonym analogies they have a tendency to opt for a high semantic associate of the incorrect context (in other words, the context consistent with the *a* and *b* analogy terms), but this context bias is frequently overcome when priming is effective. These findings provide evidence that priming may help participants overcome a bias with selecting the high semantic associate consistent with the analogy problem and may also increase a participant’s ability to inhibit the context set by the *a* and *b* terms when interpreting the homonym. From Experiment 2b it seems that falsely recalling a non-presented critical item is linked to a more efficient ability to inhibit the incorrect context of the analogy and to select the correct context item in analogical reasoning. This is consistent with the idea that false memory primes are particularly effective at priming problem-solving tasks (more so than true primes), such that they have the strength to enable inhibition of even a dominant context in problem solving.

The present findings also extend the efficacy of false priming in terms of the time taken to solve analogical reasoning problems. Previous research has demonstrated that false memory priming results in problems being solved more quickly than those that are unprimed, but these results are confined to analogies whose solutions can be easily generated via spreading activation (Howe et al., 2013). What the evidence here suggests is that even for problems whose solutions may not be as easily generated via spreading activation (at least to near associates) their solutions can also be primed by false memories. Moreover, these results show that there are significant individual differences in participants’ solution times such that a subset of participants complete analogical reasoning problems with a speed that leaves little room for any possible improvement provided by priming (our *fast solvers* subset). Given that the mean solution time for the *fast solvers* is approximately 8 seconds, which includes reading the analogy as well as the four response items, it is unlikely that the analogies could be completed more rapidly than this, leaving little room for priming effects. However, there is a subset of participants (our *slow solvers*) where false memory priming does improve solution times to levels comparable to that of fast solvers, demonstrating the efficacy of false memory priming in homonym analogy problems.

## General discussion

Previous research has established that false memories can have salutary effects (Howe, 2011; Schacter et al., 2011). One positive effect concerns the ability of false memories to prime solutions on problem-solving tasks involving insight-based reasoning (i.e., CRATs; see Howe et al., 2010b, 2011; Garner & Howe, 2014). Indeed, false memories have proved to be more effective primes for CRAT solutions than true memories when a delay (e.g., 1 week) has been imposed between the time participants recall words from studied lists and the time they are presented with CRAT problems (see Howe et al., 2012). Previous research has also demonstrated that simple verbal analogy problems can be primed using false memories but not true ones (Howe et al., 2013).

However, these findings have been restricted to conditions in which priming effects may have occurred as a result of simple spreading activation through local and highly interconnected semantic associates. This is of particular concern for studies examining analogical reasoning (Howe et al., 2013) because the problems used there may not have required analogical reasoning per se. That is, the problems could have been solved using simple associations involving BAS from the *a*, *b*, and *c* terms to the solution *d* term. This means that participants would not have had to understand the *a* to *b relation* in order to solve the problem.

The novel contribution of this series of experiments, extending our understanding of the adaptive consequences of false memories, is that false memory priming occurs even in the absence of obvious associative relations among items. That is, the current experiments made it more difficult to use only spreading activation through semantic associations to solve analogical reasoning problems by eliminating BAS within the analogy (Experiment 1) or by using homonym analogies (Experiments 2a and 2b). The findings across these three experiments provide evidence that false memories are effective primes for solutions on analogical reasoning tasks even when those solutions may not rely heavily (or perhaps at all) on spreading activation through semantic associative networks. That is, we have demonstrated for the first time that self-generated false memories can and do prime solutions to problem-solving tasks—verbal proportional analogies—in which the BAS of the analogy terms was limited so that participants had to rely on reasoning using the *a* to *b* relation to generate the *c* to *d* solution.

Furthermore, we have developed a novel set of analogies (termed *homonym analogies*) that require the inhibition of semantic associates provided by the context of the analogy in order to generate a logical (relational) solution to the analogical reasoning problem. Although existing knowledge based interpretation of homonyms might influence responses to these analogies, responses are primarily guided by interpreting the homonym in terms of the context provided by the *a*, *b*, and *c* terms of the analogy. False memory priming of the correct solution facilitated participants’ analogical reasoning not only in terms of solution rates but also in how quickly solutions were achieved (at least for participants who were not already at or near ceiling). Interestingly, when participants make errors on these analogies they do so with items that would have been generated via spreading activation through semantic associates. Of course, it is premature to conclude that adults’ (like children’s) default analogical reasoning heuristic is a search through associative networks, ones created by the biasing context of the homonym analogy itself. However, it is clear that false memory priming facilitates analogical reasoning either through the inhibition of this initial biasing context or through refocusing the search for a solution to networks related to the false memory (or both).

Indeed, our findings do not rule out the idea that adults may still use spreading activation through semantic associative networks to solve analogical reasoning problems. Although the two interpretations of the homonym (one provided by the false memory that has been generated and the other by the biasing context of the analogy) may not be compatible inasmuch as they are not ‘located’ in the same semantic neighborhoods, both interpretations will be active in associative memory at the same time. Of course, that two disparate interpretations of the same concept are active in memory at the same time is not unheard of and in some circumstances is fully anticipated (e.g., Brainerd, Wang, & Reyna, 2013; Nelson et al., 2003, 2013).

However, the question remains as to how adults reconcile these two interpretations, inhibit the more recent contextual bias from the analogy, and supplant it with the solution from the older false memory context. One likely possibility is that participants use the *a* to *b* relation to search for an alternative interpretation that is the solution to the analogy. Furthermore, it could be that the increased activation of a concept, through a process such as spreading activation, leads to increased fluidity of the concept, enabling its use in solving homonym analogies regardless of the context or interpretation. It is important to note that this increased activation and accessibility is limited to situations in which the critical lure is produced during recall; those circumstances in which the lure is not falsely recalled produce no beneficial priming effect in standard or homonym analogies. Critical lures that have been activated during DRM list presentation are still above threshold activation levels and remain highly active in memory when participants are solving the analogies, making them more accessible as a solution. Therefore, when performing a search for an alternative interpretation for a homonym analogy, false memory priming works because this alternative interpretation of the homonym is already active in memory, making this search process less difficult. Critical lures that were activated but not falsely recalled are thought to have either dropped below the activation threshold required for priming after being rejected or inhibited during test, or to have not been activated sufficiently above the threshold required for priming during study, thus reducing their accessibility when interpreting the meaning of a homonym analogy.

Whether homonym analogies are solved by applying the *a* to *b* relation to the *c* term to the generate *d* term or by searching the two distant neighborhoods of semantic associates that were recently activated for the homonym (or, indeed, by some combination of both) must await further research. However, the importance of the current results is that regardless of whether adults use semantic search, analytic mapping processes, or both, false memories can have some very positive effects inasmuch as they provide a powerful priming mechanism for solving problems.

These findings are not just important from a theoretical perspective; they carry with them some interesting everyday ramifications. This is because, as mentioned earlier, false memories occur frequently in a number of different contexts, both in and out of the laboratory (see Brainerd & Reyna, 2012; Howe, 2013). Often, these semantically activated false memories arise spontaneously and automatically, outside of the rememberer’s conscious awareness. In the world outside of the laboratory, perhaps the best known consequences of these false memories are those that have given rise to courtroom allegations of offences that may never have occurred (Howe, 2013; Schacter & Loftus, 2013). Indeed, as shown in the current research, false memories can and do serve as the basis for reasoning and decision making, and arguably do so not just in the laboratory context but in any number of everyday contexts. For example, continuing with the forensic theme, it is not just complainants’ false memories that can lead to decisions to prosecute; jurors’ false memories can lead to potential miscarriages of justice. Seminal work by Pennington and Hastie (1986, 1990, 1991, 1992) has shown that jurors activate story schemas based on their attempts to understand and integrate trial evidence. These schemas not only serve an organizing function but also serve to bias additional pieces of evidence as the trial proceeds. Worse, jurors can form false, schema-consistent memories for “facts” that are not actually in evidence. Because jury deliberations involve reasoning from such (biased) evidence, decision making as to a complainant’s guilt or innocence will be influenced not just by correct recollections of the evidence but also by (false) memories of facts not in evidence, ones that were semantically activated when the juror’s story schema was invoked.

The role of false memories in jury decision making is made more ominous given that trials usually involve considerable negative emotional content (e.g., sadness, anger, fear; see Nuñez, Schweitzer, Chai, & Myers, in press). The evidence reviewed earlier (e.g., Howe et al., 2010a) shows that negative false memories not only persist over time but can also increase over a retention interval (e.g., during the course of a trial). This is thought to be due to negative information being more densely interrelated than other types of information (Talmi, Luk, McGarry, & Moscovitch, 2007), which in turn makes spreading activation more likely through such associative networks. Indeed, previous laboratory-based research has shown that negative false memories serve as better primes than neutral false memories during an insight-based problem-solving exercise (e.g., Garner & Howe, 2014).

What these observations suggest is that because false memories can play a role in everyday cognition, including reasoning and decision making, there is a need to study their influence, both in controlled laboratory conditions as well as in more naturalistic settings. Indeed, studies have shown that false memories not only serve as powerful primes in children’s and adults’ reasoning tasks (e.g., Howe et al., 2011, 2013), some of which are used to assess intelligence and creativity, but they also play a key role in tasks frequently used to assess more perceptual components of intelligence (e.g., perceptual closure tasks; see Otgaar, Howe, van Beers, van Hoof, & Bronzwaer, 2015). Understanding the pivotal role false memories play in remembering the past, interpreting the present, and planning for the future (see Howe, 2011; Schacter et al., 2011) is essential if we are to have a complete picture of the importance of memory in everyday cognition.

## Appendix A

### Experiment 1: Analogies and Associated DRM Lists

#### Group A Analogies and DRM Lists (with BAS)

wash : clean :: press : *iron*

car : roundabout :: moon : *earth*

peace : dove :: courage : *lion*

egg : yolk :: plum : *stone*

*iron*: ore, steel, metal, crease, starch, steam, wrinkle, rust, copper, calcium, element, magnet (.10)

*earth*: planet, world, geology, ground, gravity, environment, worm, heaven, sphere, globe, core, atmosphere (.15)

*lion*: tiger, circus, jungle, tamer, den, cub, Africa, mane, cage, feline, roar, fierce (.16)

*stone*: pebble, rock, granite, kidney, sapphire, gem, brick, statue, marble, gravel, stick, tomb (.14)

#### Group B Analogies and DRM Lists (with BAS)

leopard : spots :: chest : *hair*

four : cat :: eight : *spider*

watch : cog :: compass : *needle*

prevent : restrict :: enable : *allow*

*hair*: strand, brush, scalp, lice, conditioner, comb, shampoo, headband, dandruff, mousse, bald, clippers (.31)

*spider*: web, insect, bug, fright, fly, arachnid, crawl, tarantula, poison, bite, creepy, feelers (.19)

*needle*: thread, pink, eye, sewing, sharp, point, prick, thimble, haystack, thorn, hurt, injection (.20)

*allow*: permit, let, permission, forbid, disallow, forbidden, prohibit, accept, admit, ban, admission, deny (.10)

## Appendix B

**Table 5:** Analogies normed in Experiment 2a

Analogy (*solution*)	MC foils		
	Correct context	Incorrect context high associate	Incorrect context low associate
run : legs :: stitch : *needle*	cloth	exercise	pain
table : surgery :: organ : *music*	piano	heart	donor
roar : lion :: horn : *car*	race	rhino	Africa
weapon : gun :: bug : *spider*	phobia	spy	deception
vowel : letter :: capital : *city*	address	alphabet	number
engrave : wood :: log : *book*	story	tree	branch
alive : dead :: wake : *sleep*	peace	funeral	grave
February : month :: date : *fruit*	cocktail	calendar	schedule
starve : eat :: fast : *slow*	still	food	snack

*Note*. MC = Multiple choice.

## Appendix C

### Experiment 2b Analogical Problems and DRM Lists

#### Group A Analogies (with multiple-choice A, B, and C errors, respectively) and DRM Lists (with BAS)

weapon : gun :: bug : *spider* (*phobia*, *spy*, *deception*)

starve : eat :: fast : *slow* (*still*, *food*, *snack*)

February : month :: date : *fruit* (*cocktail*, *schedule*, *calendar*)

run : legs :: stitch : *needle* (*cloth*, *exercise*, *pain*)

engrave : wood :: log : *book* (*story*, *tree*, *branch*)

*spider*: web, insect, bug, fright, fly, arachnid, crawl, tarantula, poison, bite, creepy, feelers (.19)

*slow*: fast, lethargic, stop, listless, snail, cautious, delay, traffic, turtle, speed, wait, sluggish (.17)

*fruit*: apple, vegetable, orange, kiwi, citrus, ripe, pear, banana, berry, cherry, basket, juice (.25)

*needle*: thread, pink, eye, sewing, sharp, point, prick, thimble, haystack, thorn, hurt, injection (.20)

*book*: text, library, chapter, novel, publisher, author, literature, reader, page, magazine, read, title. (.52)

#### Group B Analogies (with multiple-choice A, B, and C errors, respectively) and DRM Lists (with BAS)

vowel : letter :: capital : *city* (*address*, *alphabet*, *number*)

arithmetic : calculator :: rule : *king* (*castle*, *measure*, *depth*)

table : surgery :: organ : *music* (*piano*, *heart*, *donor*)

roar : lion :: horn : *car* (*race*, *rhino*, *Africa*)

alive : dead :: wake : *sleep* (*peace*, *funeral*, *grave*)

*city*: town, crowded, state, slum, streets, subway, country, New York, village, metropolis, big, Chicago (.17)

*king*: queen, England, crown, prince, George, dictator, palace, throne, chess, subjects, monarch (.25)

*music*: sound, harp, sing, radio, band, melody, stereo, concert, instrument, symphony, jazz, rhythm. (.24)

*car*: truck, bus, train, automobile, vehicle, drive, jeep, Ford, keys, garage, highway, van (.35)

*sleep*: rest, awake, bed, tired, dream, snooze, blanket, doze, slumber, snore, nap, yawn, drowsy (.46)

Non-critical distractor analogies:

(non-homonym distractor) caterpillar : tadpole :: butterfly : *frog* (*cocoon*, *monarch*, *moth*)

(non-homonym distractor) weep : sad :: laugh : *happy* (*cheer*, *joke*, *silly*)

(homonym distractor) fur : bear :: bark : *tree* (*leaf*, *orchard*, *log*)

## Footnotes

1 In fact, when participants were later asked during debriefing whether they thought the two tasks were in any way related, over 95 % say that they did not believe that the two tasks were connected.

2 Solution rates were examined separately for only the analogies in which the answer context corresponded with the dominant context of the homonym. A repeated measures ANOVA revealed that there was no significant main effect of priming on solution rates, *F*(2, 58) = 1.71, *p* > .05, for these analogies.

3 It is important to note that it was not possible to conduct analyses to determine if the type of priming (unprimed vs. primed no false memory vs. primed false memory) influenced the type of errors (correct context associate vs. incorrect context high associate vs. incorrect context low associate) for fast and slow solvers because there were few instances where participants were primed (and either produced or did not produce a false memory) and did not produce a correct response to the corresponding analogy item.
